# Targeted Gene Expression in Zebrafish Exposed to Chlorpyrifos-Oxon Confirms Phenotype-Specific Mechanisms Leading to Adverse Outcomes

**DOI:** 10.1007/s00128-016-1798-3

**Published:** 2016-04-16

**Authors:** Natàlia Garcia-Reyero, Lynn Escalon, Eva Prats, Melissa Faria, Amadeu M. V. M. Soares, Demetrio Raldúa

**Affiliations:** US Army Engineer Research and Development Center, 3909 Halls Ferry Rd, Vicksburg, MS 39180 USA; Institute for Genomics, Biocomputing & Biotechnology, Mississippi State University, 2 Research Blvd, Starkville, MS 39759 USA; CIC-CSIC, Jordi Girona 18, 08034 Barcelona, Spain; Department of Biology and CESAM, University of Aveiro, Aveiro, Portugal; IDAEA-CSIC, Jordi Girona 18, 08034 Barcelona, Spain

**Keywords:** Zebrafish, Gene expression, Oxidative stress, Mitochondria, Chlorpyrifos-oxon

## Abstract

Zebrafish models for mild, moderate, and severe acute organophosphorus poisoning were previously developed by exposing zebrafish larvae to chlopyrifos-oxon. The phenotype of these models was characterized at several levels of biological organization. Oxidative stress and mitochondrial dysfunction were found to be involved in the development of the more severe phenotype. Here we used targeted gene expression to understand the dose-responsiveness of those two pathways and their involvement on generating the different zebrafish models. As the severe phenotype is irreversible after only 3 h of exposure, we also analyzed the response of the oxidative stress pathway at 3 and 24 h. Some of the genes related to oxidative stress were already differentially expressed at 3 h. There was an increase in differentially expressed genes related to both oxidative stress and mitochondrial function from the more mild to the more severe phenotype, suggesting the involvement of these mechanisms in increasing phenotype severity. Temporal data suggest that peroxynitrite leading to lipid peroxidation might be involved in phenotype transition and irreversibility.

Organophosphorus compounds (OP) are acetylcholinesterase (AChE) inhibitors used primarily in pest control. There are over 3 million cases of severe acute OP poisoning (OPP) reported annually, resulting in 300,000 deaths (Bertolote 2006). Exposure to OP inhibits AChE activity leading to the accumulation of the neurotransmitter acetylcholine (ACh), activation of ACh receptors, and overstimulation of cholinergic neurons and seizures. This leads to the release of excitatory amino acids. Those will activate *N*-methyl-d-aspartate (NMDA) receptors. This results in an intracellular influx of Ca^2+^, which can cause cell damage, necrosis, and apoptosis (Brookes 2004; Pena-Llopis 2005; Kaur et al. 2014).

Organophosphate exposure has also been linked to honey bee and *Daphnia* mortality, toxicity in fish, deterioration of macroinvertebrate communities, and potential reduction in fish population growth (Southam et al. 2011; Echeverría-Sáenz et al. 2012; Zhu et al. 2014; Macneale et al. 2014; Calatayud-Vernich et al. 2016). It is therefore important to better understand the effects and mechanisms of action of OPP to better estimate hazards to organisms and populations.

A zebrafish (*Danio rerio*) model for mild, moderate, and severe OP poisoning has been previously documented (Faria et al. 2015). In order to develop the model, zebrafish larvae were exposed to increasing concentrations of chlorpyrifos-oxon (CPO), the active metabolite of the pesticide chlorpyrifos and a prototypic OP compound. The mild (P1) phenotype was characterized by behavioral impairment, which correlated with AChE inhibition. The moderate phenotype (P2) exhibited hypercontracture of the axial muscle fibers. The severe phenotype (P3), irreversible after only 3 h of exposure to CPO, was characterized by necrosis at the central nervous and neuromuscular systems. Interestingly, P3 was the only phenotype that presented a dysregulation of Ca^2+^ homeostasis, oxidative stress, and disruption of mitochondrial structure and function (Faria et al. 2015).

In order to understand the changes that triggered the transition from one phenotype to the next, we analyzed expression changes from a set of genes involved in mitochondrial function and oxidative stress in all three phenotypes. We also explored the expression of oxidative stress-related genes at 3 and 24 h on P3 embryos to explore the potential changes leading to the irreversibility of that phenotype.

## Materials and Methods

*Exposures* Embryos from wild-type zebrafish were obtained by natural mating and maintained in fish water at 28.5°C. Larvae were not fed during the entire experimental period. All procedures were conducted in accordance with institutional guidelines and approved by the Institutional Animal Care and Use Committee at the Research and Development Centre of the Spanish Research Council (CID-CSIC). The stability of CPO (Chem Service, 98.1 % purity) in fish water under exposure conditions was tested by LC–MS/MS and has been published elsewhere (Faria et al. 2015). Briefly, 7 days post-fertilization (dpf) zebrafish larvae were exposed to nominal concentrations of 0.1, 1, and 3 μM CPO in fish water, with n = 3 replicates per treatment. Control larvae were exposed under identical conditions to the same concentration of the carrier (0.1 % DMSO). Water samples were collected at 0 and 24 h of exposure and immediately analyzed using a Luna C18 (150 mm × 2 mm ID, particle size 5 μm, Phenomenex, Torrance, CA) equipped with a Security Guard pre-column. Measured CPO concentrations in water were 0, 0.097, 0.995, and 2.99 μM at 0 h of exposure and 0, 0.038, 0.684, and 1.99 μM after 24 h of exposure. Hydrolysis of CPO in water has been extensively studied, with a half-life dependent on the initial concentration (Jacobson et al. 2010). The uptake of CPO would also be responsible of a decrease of water concentration. Criteria used to classify the severity of OPP in zebrafish larvae has already been published elsewhere (Faria et al. 2015).

*RNA Extraction* Total RNA was isolated from pools of 5 larvae after thorough homogenization using a NucleoSpin RNA XS kit (Macherey–Nagel, GmbH & Co. KG, Düren, Germany) following the manufacturer’s recommendations. RNA quantity and quality were analyzed using a Bioanalyzer (Agilent Technologies, Santa Clara, CA) and a Nanodrop ND-1000 spectrophotometer (Nanodrop Technologies, Wilmington, DE). Only RNA considered to be of good quality (RIN > 8) was used for real-time PCR analysis.

*Real-Time PCR* The RT2 Profiler Zebrafish PCR Arrays for genes related to mitochondria (catalog number PAZF-087Z) and oxidative stress (catalog number PAZF-065Z) were obtained from Qiagen (Valencia, CA) in a 384-well format, which included 4 replicates of 84 genes plus standard controls (housekeeping genes, reverse transcription controls and positive PCR controls). Four biological replicates were used per treatment. The real-time PCR (RT-PCR) assays were performed on an ABI Sequence Detector 7900 (Applied Biosystems, Foster City, CA). Briefly, a total of 5 μg RNA was used to synthesize cDNA with the RT2 First Strand Kit (Qiagen, Valencia, CA) following the manufacturer’s protocol. The RT2 SYBR Green Mastermix (Qiagen, Valencia, CA) was used for RT-PCR, following the Qiagen protocol for 384 well plates containing 4 replicates of 96 assays. Cycling parameters were 95°C for 10 min, 40 cycles of 95°C for 15 s and 60°C for 1 min. The GeneGlobe Data Analysis Center (Qiagen, Valencia, USA) was used to analyze the data using the ΔΔCt method (*p* < 0.05) with normalization of the raw data to 5 different housekeeping genes. Only differentially expressed genes are included in the results.

## Results and Discussion

**Fig. 1:**
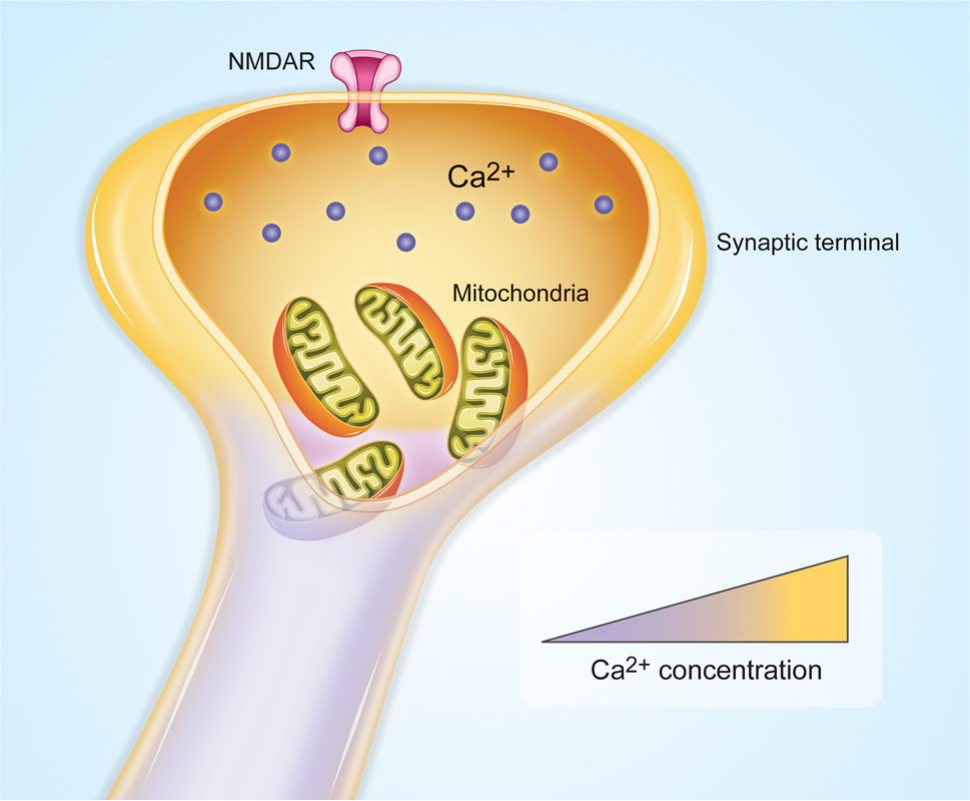
In neurons, mitochondria localized in proximity of Ca^2+^ channels such as NMDA receptors (NMDAR) accumulate Ca^2+^ and prevent the spreading of a cytosolic wave. From Rizzuto et al. (2012)

The NMDA receptors are ion channels activated by excitatory amino acids after their release due to OP-induced seizures. In neurons, mitochondria localized in proximity of the NMDA receptors, accumulate Ca^2+^ and prevent the spreading of a cytosolic wave (Rizzuto et al. 2012) (Fig. 1). The continuous activation of these receptors will lead to increased intracellular calcium, which will eventually result in mitochondrial membrane depolarization, dysregulation of mitochondrial Ca^2+^ homeostasis, production of reactive nitrogen and oxygen species (RNS and ROS), and cellular toxicity (Dong et al. 2009).

**Fig. 2:**
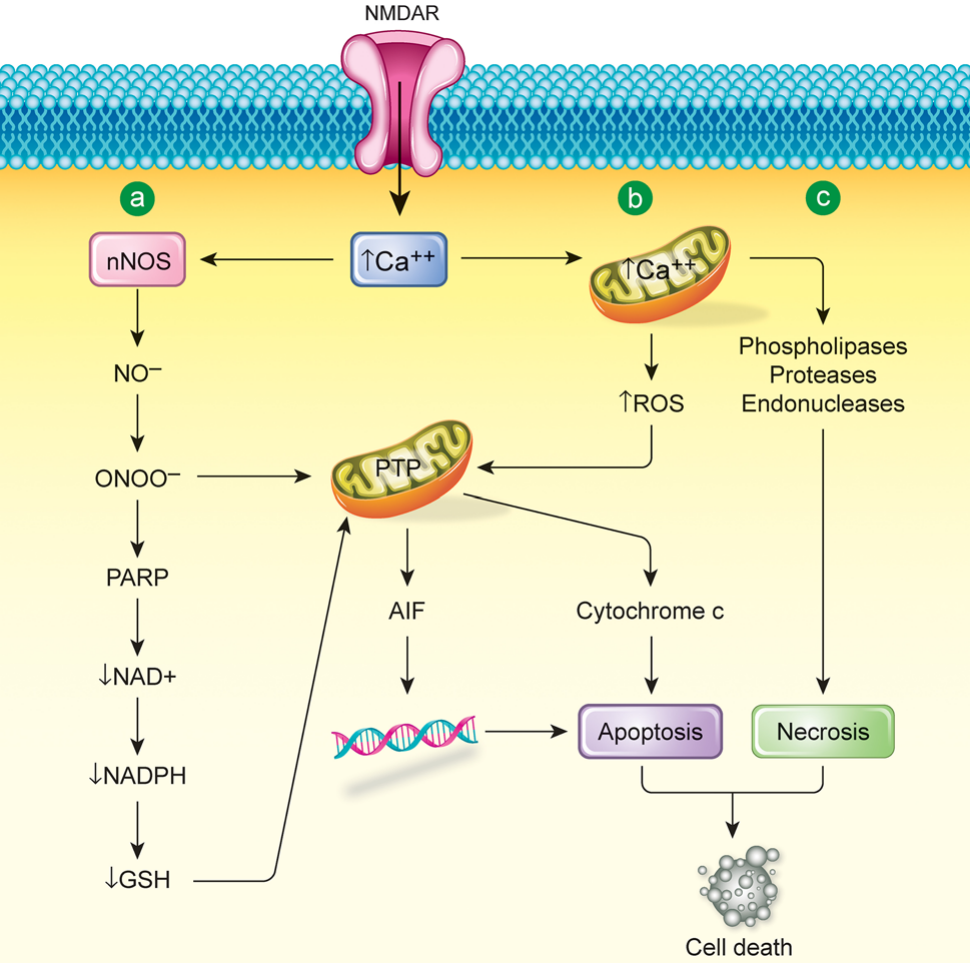
Activation of *N*-methyl-d-aspartate (NMDA) receptors can result in an intracellular influx of Ca^3+^, which can lead to *a* RNS, peroxy nitrite, *b* ROS production leading to apoptosis, *c* necrosis, and eventually cell death. From Kritis et al. (2015)

Mitochondria are the center of cellular energy metabolism, where most ATP synthesis occurs stimulated by Ca^2+^, a key regulator of mitochondrial function. Dysregulation of mitochondrial Ca^2+^ homeostasis can lead to the generation of ROS, triggering the permeability transition pore (PTP), cytochrome c release, and eventually apoptosis (Brookes 2004). The release of cytochrome c begins with its dissociation from its binding site, which increases its level in the intermembrane space. Cytochrome c is subsequently released by pore formation mediated by the pro-apoptotic Bcl-2 proteins, which eventually leads to apoptosis (Orrenius et al. 2007). The intracellular calcium overload can also result in stimulation of Ca^2+^-dependent catabolic enzymes, such as phospholipases, proteases and endonucleases that will induce necrosis (Orrenius et al. 2015) (Fig. 2).

In addition, the activation of NMDA receptors and the increase of Ca^2+^ influx lead to the production of nitric oxide levels through the activation of the nitric oxide synthase (NOS) (Kritis et al. 2015). Elevated nitric oxide (NO) concentrations can result in the production of the oxidant species peroxynitrite anion (ONOO^−^), which causes lipid peroxidation, persistent inhibition of cytochrome c oxidase, and DNA damage in neurons leading to poly(ADP-ribose) polymerase -1 (Parp1) activation. The activation of Parp1 however, only contributes to damage when it is sufficient to significantly deplete NAD^+^ (Moncada and Bolanos 2006).

As reported earlier, P3 was characterized by widespread necrosis, generation of ROS, dysregulation of the antioxidant defense system, lipid peroxidation, and mitochondrial damage including strong reduction of mitochondrial respiration. None of those effects were measurable in either P1 or P2 phenotypes (Faria et al. 2015). By exploring the regulation of genes related to oxidative stress and mitochondrial function in all phenotypes, we aimed to understand the mechanisms that triggered the transition to the more adverse phenotype.

A total of 50 genes related to oxidative stress (Tables 1 and 2) and 60 related to mitochondrial function (Table 3) were tested by RT-PCR in all three phenotypes. Unannotated genes or genes that did not pass the Qiagen standard quality control were removed from the analysis. In agreement with the phenotype severity gradient, P3 had the highest number of differentially expressed genes, followed by P2, and finally P1 had the lowest number of changes. Interestingly, most of the genes were up-regulated, particularly at P3, suggesting that somehow the organisms might have been trying to recover mitochondrial function.

**Table 1:**
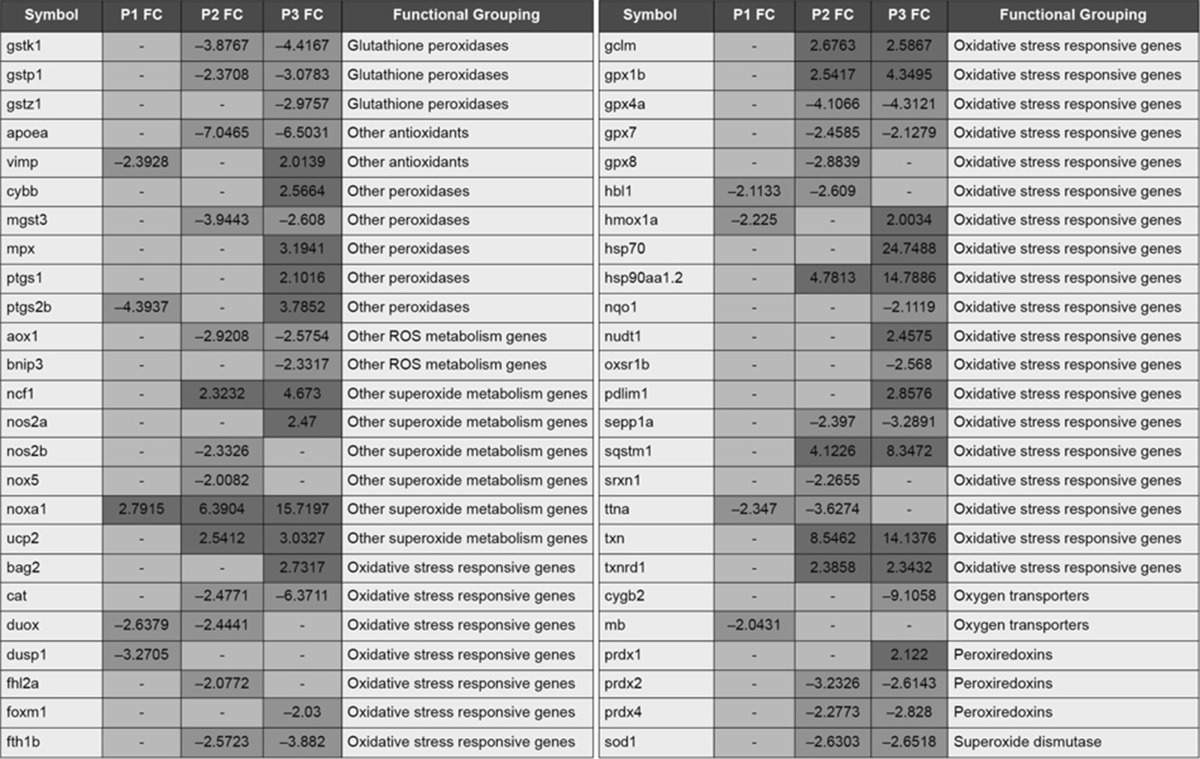
RT-PCR results for the P1 (Grade 1), P2 (Grade 2) and P3 (Grade 3) embryos at 24 h for genes related to oxidative stress (Color table online)

Results are expressed as fold change (FC). Significantly expressed genes (*p* < 0.05) are highlighted in *red* (up-regulated), or *blue* (down-regulated). Unchanged genes are highlighted in *yellow*. Gene list: aldehyde oxidase (*aox*), apolipoprotein Ea (*apoea*), BCL2-associated athanogene 2 (*bag2*), BCL2/adenovirus E1B interacting protein 3 (*bnip3*), catalase (*cat*), cytochrome b-245, beta polypeptide (*cybb*), cytoglobin 2 (*cygb2*), dual oxidase (*duox*), dual specificity phosphatase 1 (*dusp1*), forkhead box M1 (*foxm1*), ferritin heavy polypeptide 1a (*fth1b*), glutamate-cysteine ligase modifier subunit (*gclm*), glutathione peroxidase 1a (*gpx1a*), glutathione peroxidase 1b (*gpx1b*), glutathione peroxidase 4a (*gpx4a*), glutathione peroxidase 7 (*gpx7*), glutathione peroxidase 8 (*gpx8*), glutathione S-transferase kappa 1 (*gstk1*), glutathione S-transferase pi 1 (*gstp1*), glutathione S-transferase zeta 1 (*gstz1*), heme oxygenase (decycling) 1 (*hmox1a*), heat shock cognate 70-kd protein (*hsp70*), heat shock protein 90-alpha 2 (*hsp90aa1.2*), myoglobin (*mb*), microsomal gluthatione S-transferase 3 (*mgst3*), myeloid-specific peroxidase (*mpx*), neutrophil cytosolic factor 1 (*ncf1*), nitric oxide synthase 2a (*nos2a*), NADPH oxidase activator 1 (*noxa1*), NAD(P)H dehydrogenase quinone 1 (*nqo1*), nudix (nucleoside diphosphate linked moiety X)-type motif 1 (*nudt1*), oxidative-stress responsive 1b (*oxsr1b*), PDZ and LIM domain 1 (elfin) (*pdlim1*), peroxiredoxin 1 (*prdx1*), peroxiredoxin 2 (*prdx2*), peroxiredoxin 4 (*prdx4*), prostaglandin-endoperoxide synthase 1 (*ptgs1*), prostaglandin-endoperoxide synthase 2b (*ptgs2b*), selenoprotein P plasma 1a (*sepp1a*), superoxide dismutase 1 (*sod1*), sequestosome 1 (s*qstm1*), thioredoxin (*txn*), thioredoxin reductase 1 (*txnrd1*), uncoupling protein 2 (*ucp2*), VCP-interacting membrane selenoprotein (*vimp*)

**Table 2:**
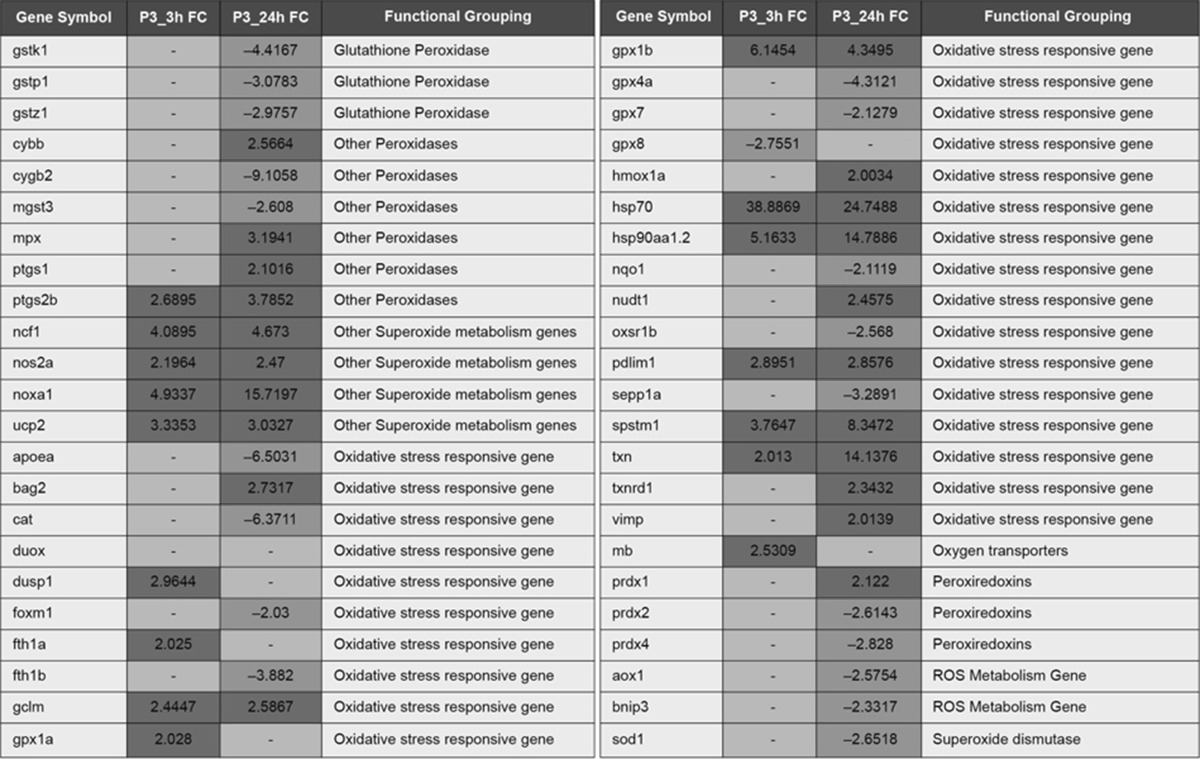
RT-PCR results for the P3 (Grade 3) embryos at 3 and 24 h for genes related to oxidative stress (Color table online)

Results are expressed as fold change (FC). Significantly expressed genes (*p* < 0.05) are highlighted in *red* (up-regulated), or *blue* (down-regulated). Unchanged genes are highlighted in *yellow*. Gene names are the same as reported in Table 1

**Table 3:**
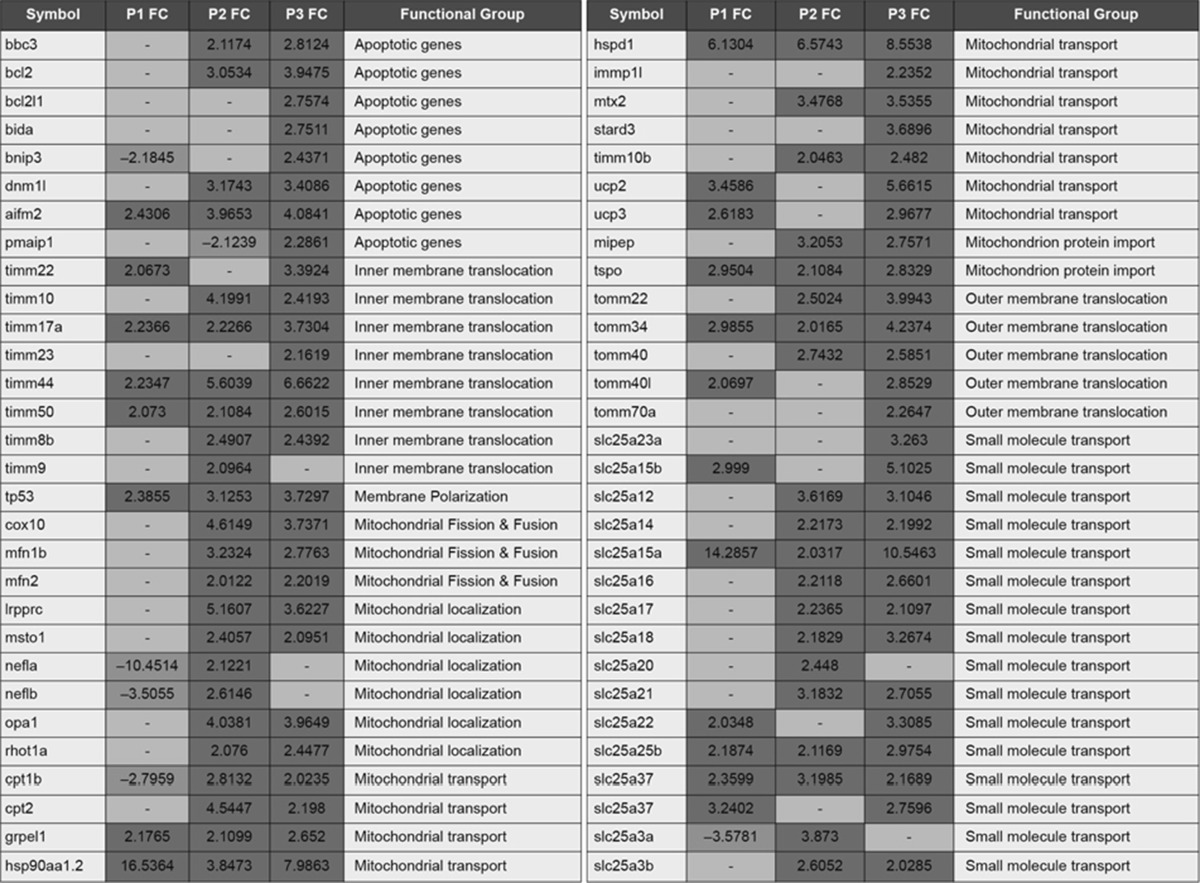
RT-PCR results for the P1 (Grade 1), P2 (Grade 2) and P3 (Grade 3) embryos at 24 h for genes related to mitochondrial function (Color table online)

Results are expressed as fold change (FC). Significantly expressed genes (*p* < 0.05) are highlighted in *red* (up-regulated), or *blue* (down-regulated). Unchanged genes are highlighted in *yellow*. Gene list: BCL2 binding component 3 (*bbc3*), B cell leukemia/lymphoma 2 (*bcl2*), Bcl2-like 1 (*bcl2l1*), BH3 interacting domain death agonist (*bida*), BCL2/adenovirus E1B interacting protein 3 (*bnip3*), COX10 heme A:farnesyltransferase cytochrome c oxidase assembly factor (*cox10*), carnitine palmitoyltransferase 1B (*cpt1b*), carnitine palmitoyltransferase II (*cpt2*), dynamin 1-like (*dnm1* *l*), GrpE-like 1 (*grpel1*), heat shock protein 90-alpha 2 (*hsp90aa1.2*), heat shock 60kD protein 1 (*hspd1*), IMP1 inner mitochondrial membrane peptidase-like (*immp1* *l*), translocase of inner mitochondrial membrane 22 (*timm22*), apoptosis-inducing factor mitochondrion-associated 2 (*aifm2*), solute carrier family 25 member 23a (*slc25a23a*), solute carrier family 25 member 15b (*slc25a15b*), leucine-rich pentatricopeptide repeat containing (*lrpprc*), mitofusin 1b (*mfn1b*), mitofusin 2 (*mfn2*), mitochondrial intermediate peptidase (*mipep*), misato homolog 1 (*msto1*), metaxin 2 (*mtx2*), neurofilament light polypeptide-like a (*nefla*), neurofilament light polypeptide-like (*neflb*), optic atrophy 1 (*opa1*), phorbol-12-myristate-13-acetate-induced protein 1 (*pmaip1*), Ras homolog gene family, member T1a (*rhot1a*), solute carrier family 25 member 12 (*slc25a12*), solute carrier family 25 member 14 (*slc25a14*), solute carrier family 25 member 15a (*slc25a15a*), solute carrier family 25 member 16 (*slc25a16*), solute carrier family 25 member 17 (*slc25a17*), solute carrier family 25 member 18 (*slc25a18*), solute carrier family 25 member 20 (*slc25a20*), solute carrier family 25 member 21 (*slc25a21*), solute carrier family 25 member 22 (*slc25a22*), solute carrier family 25 member 25b (*slc25a25b*), solute carrier family 25 member 27 (*slc25a27*), solute carrier family 25 member 37 (*slc25a37*), solute carrier family 25 member 3a (*slc25a3a*), solute carrier family 25 member 3b (*slc25a3b*), START domain containing 3 (*stard3*), translocase of inner mitochondrial membrane 10 (*timm10*), translocase of inner mitochondrial membrane 10b (*timm10b*), translocase of inner mitochondrial membrane 17a (*timm17a*), translocase of inner mitochondrial membrane 23 (*timm23*), translocase of inner mitochondrial membrane 44 (*timm44*), translocase of inner mitochondrial membrane 50 (*timm50*), translocase of inner mitochondrial membrane 8b (*timm8b*), translocase of inner mitochondrial membrane 9 (*timm9*), translocase of outer mitochondrial membrane 22 (*tomm22*), translocase of outer mitochondrial membrane 34 (*tomm34*), translocase of outer mitochondrial membrane 40 (*tomm40*), translocase of outer mitochondrial membrane 40 like (*tomm40* *l*), translocase of outer mitochondrial membrane 70a (*tomm70a*), tumor protein p53 (*tp53*), translocator protein (*tspo*), uncoupling protein 2 (*ucp2*), uncoupling protein 3 (*ucp3*)

*Oxidative Stress* The enzymes *nos2a* and *noxa1*, both involved in the formation of peroxynitrite, were upregulated in P3. This result is consistent with the presence of lipid peroxidation, increased reactive nitrogen (RN) levels, and decreased GSH levels. Interestingly, *nos2b* was downregulated only in P2 and unchanged in P3. The *noxa1* was upregulated in all three phenotypes, suggesting that recovery mechanisms were sufficient in P1 and P2 to deal with oxidative stress. Furthermore, the data suggests that the peroxynitrite route (Fig. 2a) was not significantly active in P1 and P2, as no presence of lipid peroxidation was found and only a slight decrease in GSH was detected in P2. Peroxyredoxins (Prdx) reduce peroxynitrite to nitrite (Trujillo et al. 2008). Prdx1 in particular, upregulated in P3, has been shown to inhibit oxidative stress induced apoptosis (Mei et al. 2015), which could be consistent with the need to reduce routes a and b (Fig. 2) in P3. In further support of these observations, three peroxyredoxins were significantly altered in P3, two in P2, and none in P1. Catalase (*cat*) and superoxide dismutase (*sod1*), part of the antioxidant defense activity, were downregulated in P2 and P3. Several gluthathione peroxidases and transferases were significantly altered in P2 and P3, but not P1.Thioredoxin (*txn*), an enzyme involved in decreasing oxidative stress, was upregulated in P2 and P3. Necrosis (Fig. 2c) was also involved in P3 development, as shown in Faria et al. (2015).

As the more severe phenotype (P3) is reversible before 3 h of exposure, we analyzed genes relative to oxidative stress at both 3 and 24 h of exposure, in order to explore the initiating mechanisms that might lead to the permanent adverse outcome. Interestingly, *nos2a* and *noxa1* were two of the few genes significantly up-regulated at 3 h (Table 2), suggesting that the peroxynitrite route (a) might be one of the initial mechanisms leading to toxicity in the cell. Two glutathione peroxidases, as well as *txn*, were also up-regulated at the early time point, suggesting that the processes resulting in the formation of oxidative stress had already began. In addition, *hsp70* and *hsp90aa1.2*, proteins that respond to stress, were already up-regulated at 3 h.

*Mitochondrial Function* Twenty-six genes related to apoptosis and mitochondrial function were significantly expressed in P1, 46 in P2, and 55 in P3 (Table 3). The increasing number of affected genes also correlated with the increased severity of the phenotypes. The solute carrier family 25 (Slc25) proteins are carriers that facilitate the transport of solutes across the inner mitochondrial membrane. Specifically, *slc25a12* (up-regulated in P2 and P3), *slc25a23a* (up-regulated in P3), and *slc25a25* (up-regulated in all three phenotypes) are Ca^2+^-sensitive mitochondrial carriers (Gutiérrez-Aguilar and Baines 2013). In addition, *slc25a17*, up-regulated in P2 and P3, has been linked to a myopathy involving inhibition of muscular relaxation in horses, believed to be derived from an excess of calcium triggering ATP depletion (Barrey et al. 2012). These results are consistent with the reported increase in intracellular levels of Ca^2+^ after NMDA receptor activation by OPs (Faria et al. 2015).

Most of the proteins related to inner (*timm* family) or outer (*tomm* family) mitochondrial membrane transport were up-regulated in P2 (10) and P3 (12), and some in P1 (5), further suggesting that mitochondrial function and transport was not only important for phenotype development, but also increased with the severity of the outcome. All apoptotic genes were up-regulated in P3. Particularly, genes related to the pro-apoptotic Bcl-2 protein (*bbc3*, *bcl2*, *bcl21* *l*, and *bnip3*), involved in the apoptosis route (Fig. 2b) mediated by cytochrome c (Orrenius et al. 2007). This arguably indicates the potential for apoptosis leading to cell death in P3 and, in a lower amount, in P2.

In conclusion, we analyzed the expression of genes related to oxidative stress and mitochondrial function in the zebrafish model for mild, moderate, and severe OP poisoning. Our data showed that the number of genes affected increased in accordance with the severity of the phenotype. Furthermore, temporal data strongly suggest that the activation of reactive nitrogen species and peroxynitrite leading to lipid peroxidation and apoptosis might be the initial step leading to irreversibility in P3, as well as an important part of the transition from P2 to P3.
